# Gravity-Dependent Animacy Perception in Zebrafish

**DOI:** 10.34133/2022/9829016

**Published:** 2022-08-30

**Authors:** Xiaohan Ma, Xiangyong Yuan, Jiahuan Liu, Li Shen, Yiwen Yu, Wen Zhou, Zuxiang Liu, Yi Jiang

**Affiliations:** ^1^State Key Laboratory of Brain and Cognitive Science, CAS Center for Excellence in Brain Science and Intelligence Technology, Institute of Psychology, Chinese Academy of Sciences, Beijing 100101, China; ^2^University of Chinese Academy of Sciences, Beijing 100049, China; ^3^Chinese Institute for Brain Research, Beijing 102206, China; ^4^State Key Laboratory of Brain and Cognitive Science, Institute of Biophysics, Chinese Academy of Sciences, Beijing 100101, China; ^5^Institute of Artificial Intelligence, Hefei Comprehensive National Science Center, Hefei 230088, China

## Abstract

Biological motion (BM), depicted by a handful of point lights attached to the major joints, conveys rich animacy information, which is significantly disrupted if BM is shown upside down. This well-known inversion effect in BM perception is conserved in terrestrial vertebrates and is presumably a manifestation of an evolutionarily endowed perceptual filter (i.e., life motion detector) tuned to gravity-compatible BM. However, it remains unknown whether aquatic animals, living in a completely different environment from terrestrial animals, perceive BM in a gravity-dependent manner. Here, taking advantage of their typical shoaling behaviors, we used zebrafish as a model animal to examine the ability of teleosts to discriminate between upright (gravity-compatible) and inverted (gravity-incompatible) BM signals. We recorded their swimming trajectories and quantified their preference based on dwelling time and head orientation. The results obtained from three experiments consistently showed that zebrafish spent significantly more time swimming in proximity to and orienting towards the upright BM relative to the inverted BM or other gravity-incompatible point-light stimuli (i.e., the non-BM). More intriguingly, when the recorded point-light video clips of fish were directly compared with those of human walkers and pigeons, we could identify a unique and consistent pattern of accelerating movements in the vertical (gravity) direction. These findings, to our knowledge, demonstrate for the first time the inversion effect in BM perception in simple aquatic vertebrates and suggest that the evolutionary origin of gravity-dependent BM processing may be traced back to ancient aquatic animals.

## 1. Introduction

The ability to readily distinguish animate entities from inanimate objects in the environment is essential to animal survival and social interaction [1]. Biological motion (BM), represented by the movements of a few point lights attached to the major joints [2], can provide sufficient visual information for the detection and recognition of living entities. However, visual perception of BM in point-light displays (PLDs) is strongly impaired when the PLDs are presented upside down [3–14]. This phenomenon, known as the inversion effect in BM perception, presumably reflects an evolutionarily endowed sensitivity of the visual system tuned to gravity-compatible life motion signals.

Previous studies have demonstrated that the inversion effect in BM perception does not rely on postnatal experience and is widely conserved in terrestrial vertebrates, as both human newborns and visually inexperienced chicks manifest spontaneous preference for upright than inverted point-light BM signals [3, 4, 7, 11, 14]. This superior inborn ability to discriminate and recognize the spatiotemporal patterns of life motion is associated more with the dynamic rather than the configural processing of BM [13, 15]. As evidence, detection accuracy for unfamiliar upright displays, such as walking on hands, is also higher than their inverted counterparts [10]. More importantly, even when a point-light walker is spatially scrambled such that the configural information is severely destroyed and only the kinematic information remains unaltered, the perceived walking direction of the scrambled walker is almost unaffected for the upright displays, but the performance for the inverted ones drops to chance level [13]. A similar response pattern can also be found in baboons [8].

These observations encourage researchers to propose a “life motion detector” theory [13], which claims that the superior ability to detect and recognize upright BM is likely due to the innate sensitivity of the visual system tuned to the gravity-dependent dynamics of life motion signals [16], especially arising from the feet motion [3, 13, 17]. Upright feet motion can attract attention rapidly at the early stage of visual processing [18] and facilitate visual search [19]. Even newborn infants with no visual experience spend more fixation time on upright than inverted PLDs representing the legs of a walking animal [3]. All the evidence has led researchers to enrich the “life motion detector” theory by further proposing that for the terrestrial animals, when their feet are pushed off the ground and fall down under gravity, it is the ballistic velocity trajectories that provide valuable clues to the detection of life motion signals [13]. Our recent study with astronauts in spaceflight further confirms that the difference in sensitivity to the upright BM versus the inverted BM is largely shaped by the gravity of Earth [20].

The inversion effect in BM perception explained by the “life motion detector” theory has so far been observed in terrestrial animals. However, it remains largely unknown how it would be like in lower aquatic vertebrates, such as teleosts, which live in a completely different environment from terrestrial animals. In fish, the shoaling behavior affiliating with conspecifics, as an important social behavior [21], has already been widely reported to be driven by one dot placed on the centroid [22, 23] or a series of dots attached on the backbone of a swimming fish [24–26]. Compared with the non-BM stimuli that are composed of a similar number of dots as the BM stimuli but with very different motion dynamics, fish are more willing to swim near the BM stimuli [25], indicating that they are able to detect and recognize conspecifics by only motion cues. Even juvenile fish show intimate affiliation with the movement of dots that mimics fish motion, confirming that the visual sensitivity to BM may be innate for teleosts [22]. We are therefore interested in whether the inversion effect in BM perception found in the terrestrial animals essentially originates from their remote ancestors (i.e., the aquatic vertebrates). To examine this hypothesis, here we presented upright (gravity-compatible) and inverted (gravity-incompatible) BM of teleosts to zebrafish and analyzed their dwelling time and head orientation to quantify to what extent zebrafish prefer upright over inverted (and other gravity-incompatible) PLDs.

## 2. Methods

### 2.1. Fish and Housing Conditions

Adult wild-type (AB) zebrafish (*Danio rerio*) with an age range of 6−7 months were used. All fish were kept in mixed sex groups in storage tanks under a recirculation life support system, with water temperature at 28.0°C, pH 7.0, and a 14L:10D light:dark cycle. Fish older than 18 dpf were fed with *brine shrimp* every morning at about 10 am. All experiments were conducted in accordance with standard guidelines and regulations and were approved by Institute of Biophysics, Chinese Academy of Sciences.

### 2.2. Behavioral Setup

**Figure 1:**
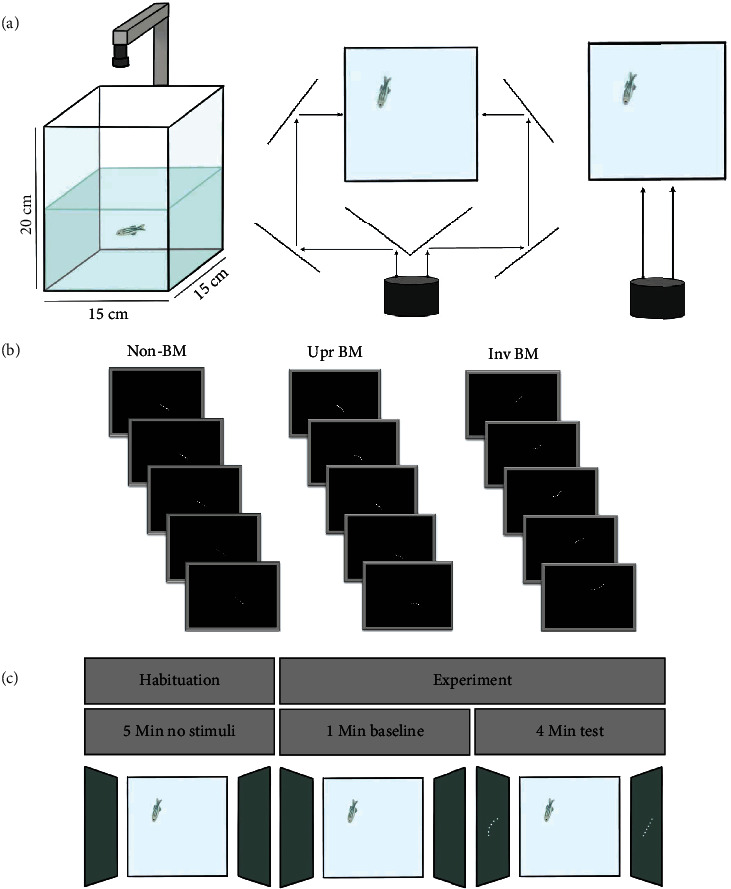
Behavioral paradigm. (a) Experimental setup. The water had a depth of 10 cm, and the infrared camera was placed just above the tank. In experiments 1 and 2, the stimuli were projected to the tank through multiple reflections of the mirrors. In experiment 3, the stimuli were projected directly to the tank. (b) Experimental stimuli. Upright BM and non-BM (or inverted BM) stimuli were represented as the animations of six white dots. (c) Experimental procedure. Upright BM and non-BM (or inverted BM) stimuli were either projected onto two opposite sides of the tank separately in a within-subject design (experiments 1 and 2) or onto one side of the tank in a between-subject design (experiment 3). The habituation period took at least 5 minutes until the fish swam in the tank in a normal pattern and speed. The experiment period included a 1-minute baseline and 4-minute test.

Cubic glass aquaria measured 15 × 15 × 20 cm was used as the test tank (Figure 1(a)). Opaque gray glass films were attached to the inside sides of the test tank to prevent external influences. The test tank was filled up with housing water to 10 cm depth. In experiments 1 and 2, the stimuli were projected onto two opposite sides of the test tank separately through an optic reflection equipment consisting of six one-way mirrors positioned on the two opposite sides of the tank, with three mirrors per side. The first mirrors received the stimuli from a projector controlled by the experimental computer and reflected them to the second and the third mirrors, which then projected them to the two sides of the tank. Compared to presenting the stimuli with two projectors or two displays, the current setup occupied a small space and was simple to implement, and more importantly, it effectively avoided the potential interference between different instruments. In experiment 3, the stimuli were directly projected onto one side of the test tank by the projector. Infrared lights illuminated the tank from the bottom to avoid the interference from the luminance changes produced by the stimuli, and an infrared-compatible camera (MV-1200, PDV, Beijing, China) placed above the tank was connected to another computer, which monitored and recorded the moving trajectories of the focal fish.

### 2.3. Stimuli

The upright BM stimuli of three female fish used in the present study were created and provided by Nakayasu et al. [25, 27] and can be downloaded from figshare (doi:10.6084/m9.figshare.5947447). The recorded stimuli consist of six dots placed at equal distance along the fish body, sampled at 60 fps rate during 1-minute (3600 frames) motion tracking of the fish freely swimming in a tank. In our experiments, the six dots of the upright BM stimuli were projected in white onto the gray glass films of the tank, and the projected full length of the stimuli was about 2.5 cm, comparable to the normal length of an adult zebrafish (Figure 1(b)). Consistent with many other studies [25, 26], the upright BM stimuli were presented solely based on the data of two-dimensional coordinates (*x*, *y*) from the side view because of the dimensional limits of a computer display. In addition to the upright BM stimuli (i.e., the gravity-compatible stimuli), we created another two stimuli as gravity-incompatible comparisons. One was the non-BM stimuli, of which the six dots were kept in a constant distance (i.e., the average distance between the dots across all frames) and moved along a linear trajectory with a constant speed (i.e., the average speed across all frames). The differences between the upright BM and the non-BM stimuli included the body configuration, swim trajectory, and swim kinetics. The other one was the inverted BM stimuli, generated by mirror flipping the original upright BM stimuli vertically. By this means, the only difference between the upright BM and the inverted BM stimuli was the swim kinetics, which was specifically reflected in the direction of the vertical motion velocity and acceleration (i.e., compatible or incompatible with the direction of gravity, see demos in Supplementary Materials).

### 2.4. Experimental Procedures

On the day before test, the fish from the storage tank was put into the test tank overnight (23 h ± 1 h). On the test day, the tank was moved to test bench, and the fish was allowed to habituate the test environment for at least 5 minutes (Figure 1(c)). After the habituation period, the behaviors of the fish were continuously recorded for a baseline period of 1 minute (no stimuli) and a test period of 4 minutes, during which the 1-minute stimuli were repeated 4 times [25]. The fish was put back into another storage tank after the experiment. Each fish had no experimental experience before the experiment and was tested only once in this study. In experiment 1, the upright BM and the non-BM stimuli were simultaneously projected onto the two opposite sides of the test tank, respectively. In experiment 2, the upright BM and the inverted BM stimuli were projected onto the two sides of the test tank as in experiment 1, while in experiment 3, they were presented on a single side of the tank, with each fish exposed to only one type of the stimuli. The presentation side of the BM stimuli and the three BM stimuli recorded from different fish were balanced across subjects in all experiments.

### 2.5. Motion Tracking

The motion trajectories of the fish in each experiment were recorded at a rate of 2 fps and saved by a custom-made video recording software. The saved data were offline imported into MATLAB (the MathWorks) and analyzed using custom-made scripts, which referred to a tracking software developed in Python (see https://github.com/joseaccruz/fshtracker). The motion tracking analysis here followed the pipelines of Abril-de-Abreu et al. [28]. First, the fish was searched for in a predefined 2D region, corresponding to a 12 × 12 cm inner area of the test tank, which guaranteed a sufficiently high contrast for separating out the fish's configuration. Second, the head, centroid, and tail of the fish within this 2D region were detected for each frame by our custom-made scripts, which also allowed a visual inspection of the tracking quality and a manual correction of possible tracking errors. Finally, the pixel coordinates of the head, centroid, and tail of each fish for each frame were saved for further determination of the position and orientation.

### 2.6. Behavioral Data Analysis and Statistics

**Figure 2:**
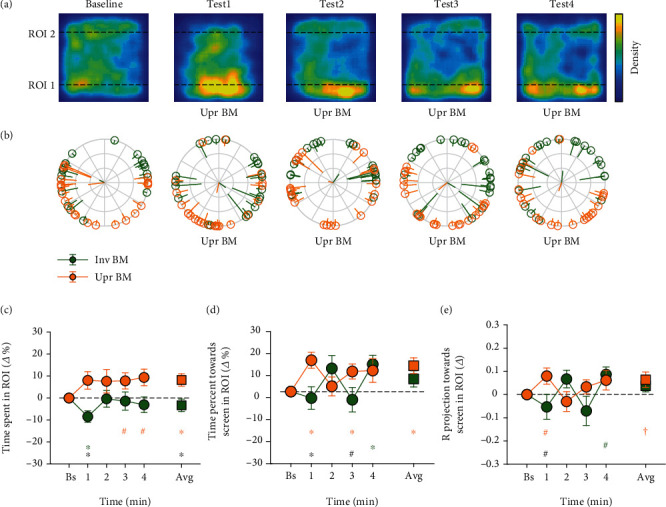
The results of experiment 1. (a) 2D heatmaps of the fish's swimming positions in each of the 1-minute time bins. The density is calculated by dividing the amount of time that the fish stayed at each position by the total amount of time in each time bin and then scaled to [0, 1]. The warmer the color, the more frequently the fish stayed at this position. The bottom side is the screen on which the upright BM stimuli were presented. (b) Average oriented direction of each fish in each ROI. Each dot on the circle represents one fish. The orange and green dots represent the fish in ROI 1 and ROI 2, respectively. The tail of the dot points to the average oriented direction of the fish across frames, with its length inversely representing the variation. The shorter the length, the larger the variation. The central two rods show the average vector of direction focus across fish in each ROI. (c) The relative percentage of time the fish spent in each ROI over separate time bins as well as the average (test minus baseline). (d) The relative percentage of time the fish oriented towards the nearby stimulus in each ROI. (e) The relative magnitude of the mean direction focus of the fish towards the nearby stimulus in each ROI (the Rproj). The circles (or squares) and the error bars represent the sample mean ± SEM. Note: ^∗^*p* < 0.05, with FDR corrected for separate time bins; ^#^*p* < 0.05, uncorrected; ^†^*p* < 0.1. The orange and green signs mark the significant differences between the test and the baseline periods for the upright BM and the non-BM stimuli, respectively, while the black ones mark the significant differences between the upright BM and the non-BM stimuli during the test period.

**Figure 3:**
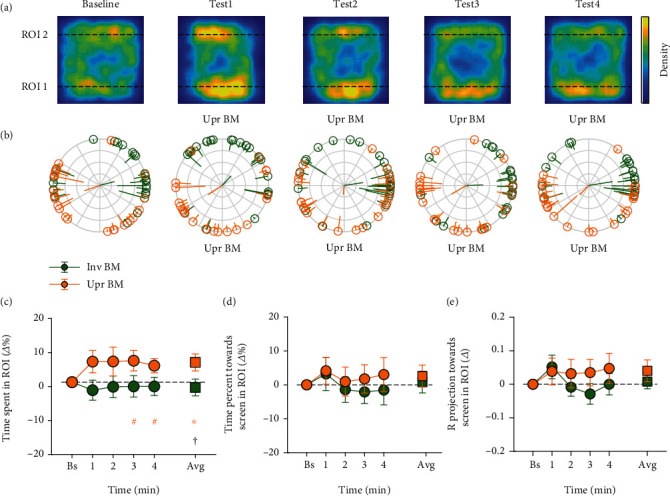
The results of experiment 2. (a) 2D heatmaps of the fish's swimming positions in each of the 1-minute time bins. The warmer the color, the more frequently the fish stayed at this position. The bottom side is the screen on which the upright BM stimuli were presented. (b) Average oriented direction of each fish in each ROI. Each dot on the circle represents one fish. The orange and green dots represent the fish in ROI 1 and ROI 2, respectively. The tail of the dot points to the average oriented direction of the fish across frames, with its length inversely representing the variation. The central two rods show the average vector of direction focus across fish in each ROI. (c) The relative percentage of time the fish spent in each ROI over separate time bins as well as the average. (d) The relative percentage of time the fish oriented towards the nearby stimulus in each ROI. (e) The relative magnitude of the mean direction focus of the fish towards the nearby stimulus in each ROI (the Rproj). The circles (or squares) and the error bars represent the sample mean ± SEM. Note: ^∗^*p* < 0.05, with FDR corrected for separate time bins; ^#^*p* < 0.05, uncorrected; ^†^*p* < 0.1. The orange signs mark the significant differences between the test and the baseline periods for the upright BM stimuli.

**Figure 4:**
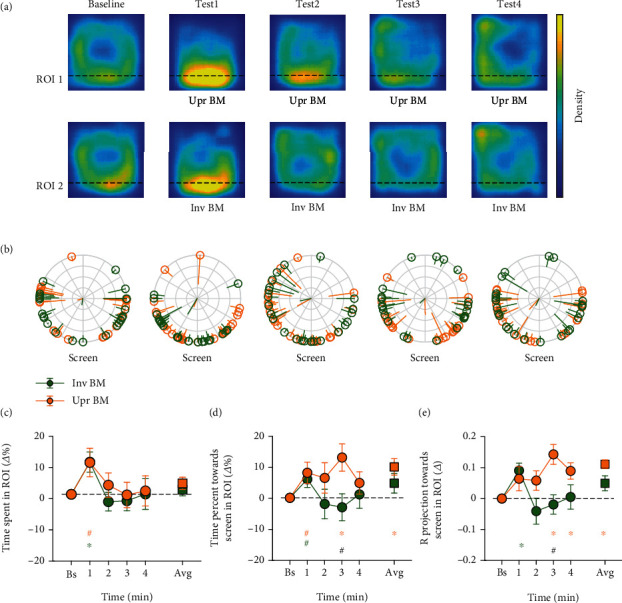
The results of experiment 3. (a) 2D heatmaps of the fish's swimming positions in each of the 1-minute time bins. The warmer the color, the more frequently the fish stayed at this position. The bottom side is the screen on which the stimuli were presented. (b) Average oriented direction of each fish in each ROI. Each dot on the circle represents one fish. The orange and green dots represent the fish in ROI 1 and ROI 2, respectively. The tail of the dot points to the average oriented direction of the fish across frames, with its length inversely representing the variation. The central two rods show the average vector of direction focus across fishes in each ROI. (c) The relative percentage of time the fish spent in each ROI over separate time bins as well as the average. (d) The relative percentage of time the fish oriented towards the nearby stimulus in each ROI. (e) The relative magnitude of the mean direction focus of the fish towards the nearby stimulus in each ROI (the Rproj). The circles (or squares) and the error bars represent the sample mean ± SEM. Note: ^∗^*p* < 0.05, with FDR corrected for separate time bins; ^#^*p* < 0.05, uncorrected. The orange and green signs mark the significant differences between the test and the baseline periods for the upright and the inverted BM stimuli, respectively, while the black ones mark the significant differences between the upright and the inverted BM stimuli during the test period.

All the data analysis below was conducted by a custom-made script in MATLAB platform. The behaviors of each fish in the baseline period were quantified to exclude the fish (1) staying within a restricted area and seldom swimming around and (2) frozen for more than 4 s. A region of interest (ROI) corresponding to an area near the stimuli with the width equal to the body length of an adult zebrafish (20% of the tank) was predefined. In all of the three experiments, the area beside the upright BM stimuli was labeled ROI 1 and the area beside the non-BM (or the inverted BM) stimuli was ROI 2 (see Figures 2(a)–4(a)).

Three dependent variables were calculated to examine the potential preference of the fish for the upright BM stimuli. The first one was the percentage of time the fish spent in each ROI (i.e., the time spent in the ROI divided by the whole time spent in the tank). The second one was the percentage of time the fish oriented towards the nearby stimulus in each ROI (i.e., the time when the fish oriented towards the stimulus within a tolerance range of ±90° in the ROI divided by the whole time they spent in the ROI). The third one measured the direction focus towards the stimulus, namely the Rproj value [28]. Briefly, for each fish, the orientation in each frame taken during the baseline and the test periods was first transformed to a unit vector and then averaged across frames in the ROI. The norm of this vector ranged from 0 to 1 was then projected onto the direction orthogonal to the nearby screen in the ROI. The Rproj ranged from -1 to 1, with positive values indicating the directionality towards the stimulus, negative values away from it, and zero no directionality. The larger the Rproj, the more concentrated (or smaller variation) the direction focus of the fish over time.

In the three experiments, the above dependent variables in each ROI were not only calculated for the baseline and the test period, but also for separate time bins, each with a duration of 1 minute (i.e., the 1st, 2nd, 3rd, and 4th minutes of the test period). To obtain more reliable estimates, the three dependent variables computed in the test period were normalized by subtracting the corresponding values in the baseline and represented by *Δ*% or *Δ* in Figures 2–4. Data normality was tested by the Shapiro-Wilk normality test. Parametric paired and independent *t*-tests were used for normally distributed data, and nonparametric permutation *t*-tests were used for nonnormally distributed data by using the PERMUTOOLS available at https://github.com/mickcrosse/PERMUTOOLS. The differences between the baseline and the test periods and the differences between the upright BM and the non-BM (or the inverted BM) conditions were statistically compared in experiments 1–3. The statistical analyses were performed using MATLAB and SPSS. All significant *p* values obtained from the comparisons of the baseline and separate time bins, if not specifically indicated, had passed the false discovery rate (FDR) correction [29, 30]. All behavioral results were represented as mean ± SD.

## 3. Results

### 3.1. Experiment 1

Experiment 1 is aimed at confirming in our experimental setup that the shoaling behavior is driven by the upright BM (gravity-compatible) stimuli rather than the non-BM (gravity-incompatible) stimuli. A total of 31 fish (16 female) were tested, with 24 qualified fish (12 female) for statistical analyses (see the exclusion criteria in Methods section, and one fish was excluded as more than 500 frames were missed due to the technical issue).

The sampled swimming positions of all fish were pooled together during the baseline and the test periods and drawn in 2D heatmaps (Figure 2(a)). The fish swam towards the upright BM rather than the non-BM stimuli after these two types of stimuli simultaneously appeared on the opposite sides of the screen during the test period. First of all, we showed that there was no difference between the percentage of time spent in ROI 1 and ROI 2 during the baseline period (16.86 ± 10.40% and 17.65 ± 13.16%, respectively; *t*_23_ = −0.228, *p* = 0.830), confirming that the fish had no prior preference for certain positions in the tank. Next, we demonstrated that the fish on average spent significantly more time in ROI 1 during the test period compared with that during the baseline (*t*_23_ = 2.738, *p* = 0.012, the orange square in Figure 2(c)). This trend started from the beginning of the test period and was more obvious in the 3rd and 4th minutes. By contrast, no significant difference of the average time spent in ROI 2 between the test and the baseline periods was found (*t*_23_ = −1.167, *p* = 0.255, the green square in Figure 2(c)), and the fish even spent less time with the non-BM stimuli in ROI 2 in the 1st minute of the test period compared with the baseline (*t*_23_ = −3.230, *p* = 0.004). When the relative time percentage spent in ROI 1 was directly compared with that in ROI 2, the results further confirmed that the fish preferred to approach the upright BM than the non-BM stimuli; i.e., they would like to spend significantly more time in ROI 1 than ROI 2 (8.21 ± 14.69% and −3.31 ± 13.89%, *t*_23_ = 2.431, *p* = 0.023). This pattern was more pronounced in the 1st minute of the test period (*t*_23_ = 3.154, *p* = 0.004).

The head orientation of each fish and the group average direction focus relative to the stimulus direction (the Rproj) were depicted in Figure 2(b). Similarly, during the baseline period, the percentage of time when the fish oriented towards the nearby stimulus (41.55 ± 17.04% and 44.57 ± 14.49%, respectively; *t*_22_ = −0.733, *p* = 0.472) and the Rproj (0.019 ± 0.151 and 0.025 ± 0.122, respectively; *t*_22_ = −0.148, *p* = 0.887) had no significant differences between the upright BM and the non-BM stimuli. We then examined whether the fish were prone to head towards the upright BM stimuli during the test period than during the baseline period. As shown in Figure 2(d), the fish spent significantly more time watching the upright BM stimuli during the whole test period (10.77 ± 16.27%, *t*_23_ = 3.241, *p* = 0.004, the orange square). By contrast, no significant difference was found in ROI 2 where the non-BM stimuli were presented during the whole test period (5.27% ± 16.39%, *t*_22_ = 1.542, *p* = 0.137, the green square). In particular, the time spent towards the stimuli significantly increased in ROI 1 from the baseline in the 1st and the 3rd minutes (the 1st minute: *t*_23_ = 3.908, *p* = 0.001; the 3st minute: *t*_22_ = 2.614, *p* = 0.016) and increased in ROI 2 in the 4th minute (*t*_20_ = 2.865, *p* = 0.010). Further comparison revealed that the time spent towards the stimuli in ROI 1 was significantly more than that in ROI 2 in the 1st minute (*t*_21_ = 3.373, *p* = 0.003). The Rproj showed a similar response pattern and time course (Figure 2(e)). The fish concentrated more on the direction towards the upright BM stimuli, as the Rproj value was marginally increased relative to the baseline (0.064 ± 0.162, *t*_23_ = 1.936, *p* = 0.065, the orange square). The results together suggest that the fish prefer to watch the upright BM stimuli rather than the non-BM stimuli.

### 3.2. Experiment 2

Consistent with previous findings [23, 25], experiment 1 demonstrated that the zebrafish are prone to the upright BM stimuli and prefer to approach and look at the upright BM stimuli especially in the first minute during the test period. Based on these results, we carried out experiment 2 to further explore whether the zebrafish are able to distinguish between the upright BM and the inverted BM stimuli simultaneously presented on the two opposite screens using the same setup. A total of 28 fish (14 female) were tested, and finally, 24 fish (12 female) were included in the following statistical analyses.

Similar to experiment 1, the fish swam freely with no preference to each ROI in the baseline period (15.55 ± 8.10% and 18.71 ± 10.60%, respectively; *t*_23_ = −1.088, *p* = 0.288; see Figure 3(a)), and they headed towards the upright BM and the inverted BM stimuli in equal probability (48.15 ± 12.80% and 47.00 ± 16.64%, respectively; *t*_22_ = 0.289, *p* = 0.775) and with equal direction focus (i.e., the Rproj: 0.048 ± 0.134 and 0.029 ± 0.107, respectively; *t*_22_ = 0.540, *p* = 0.595). In the test period, we found that the fish were more willing to swim near the upright BM stimuli (Figure 3(c)). Specifically, the fish spent more time in ROI 1 during the whole test period compared with the baseline (5.47 ± 11.46%, *t*_23_ = 2.339, *p* = 0.017, the orange square). This trend seemed to emerge once the fish saw the upright BM stimuli and became more obvious in the 3rd and 4th minutes. By contrast, the fish did not spend more time in ROI 2 during the whole test period compared with the baseline (−1.47 ± 11.47%, *t*_23_ = 0.627, *p* = 0.537, the green square). A marginally significant difference between the average time percentage the fish spent in ROI 1 and ROI 2 was found (*t*_23_ = 1.776, *p* = 0.089). However, neither did we observe significant changes of the time spent towards the stimuli and the Rproj in the test period for ROI 1 and ROI 2, nor their differences between these two ROIs (ps > 0.15, Figures 3(d) and 3(e)).

The results obtained from experiment 2 suggest that the zebrafish seem to own the ability to discriminate between the upright BM and the inverted BM stimuli and tend to stay with the upright BM stimuli for more time. But as there was no significant supportive evidence that the fish more likely orient towards the upright BM stimuli during the test period, it needs to be further verified whether the discrimination between the upright BM and the inverted BM stimuli can be robustly reflected in the head orientation of the zebrafish.

### 3.3. Experiment 3

Experiment 2 demonstrated that the zebrafish were more willing to swim near the upright BM stimuli but did not find the preference of the zebrafish to head towards the upright BM stimuli. This was likely due to the potential competition from the simultaneous presentations of these two very similar stimuli. The zebrafish would have more time exploring the stimuli in a single-display setup, which could likely increase the probability that the zebrafish orient towards the stimuli. Hence, we further tested in experiment 3 whether the head orientation is a sensitive index for the discrimination between the upright BM and the inverted BM when the stimuli were presented on a single screen for each fish. A total of 71 fish were tested, and finally, 48 fish remained in statistical analyses with half of them exposed to the upright BM stimuli (9 female) and the other half to the inverted BM stimuli (9 female).

First, the two group of fish spent a similar amount of time in ROI 1 and ROI 2 during the baseline period (21.12 ± 13.35% and 17.89 ± 10.39%, respectively; *t*_46_ = 0.937, *p* = 0.346). As depicted in Figures 4(a) and 4(c), although the average time the fish spent during the test period in both ROIs had no significant changes relative to the baseline (ps > 0.32), they swam quickly to the stimulus immediately after its appearance. The time spent increased in the 1st minute in both ROI 1 (9.53 ± 20.94%, *t*_23_ = 2.230, *p*_uncorrected_ = 0.033) and ROI 2 (9.65 ± 14.77%, *t*_23_ = 3.200, *p* = 0.004), suggesting that both stimuli are attractive to the zebrafish at the first glance. In experiment 3, we did not find significant differences between ROI 1 and ROI 2 (ps > 0.28), probably due to the reduced statistical power in a between-subject design.

The time percentage of the fish heading towards the screen (44.54 ± 11.92% and 46.97 ± 12.10%, respectively; *t*_46_ = −0.699, *p* = 0.488) and their direction focus (0.015 ± 0.098 and 0.062 ± 0.123, respectively; *t*_46_ = −1.449, *p* = 0.154) from the two ROIs did not differ from each other in the baseline period. Crucially, the fish spent more time on orienting toward the upright BM stimuli (9.87 ± 12.88%, *t*_23_ = 3.754, *p* = 0.001, the orange square in Figure 4(d)) with a significantly higher Rproj (0.111 ± 0.097, *t*_23_ = 5.571, *p* < 0.001, the orange square in Figure 4(e)) during the test period compared with the baseline. These differences went larger as time progressed, particularly in the 3rd (head orientation: *t*_23_ = 2.953, *p* = 0.007; Rproj: *t*_23_ = 3.155, *p* = 0.004) and the 4th minutes (Rproj: *t*_23_ = 2.398, *p* = 0.014), which substantiated the facts that the fish may require more time to distinguish the upright BM from the inverted BM stimuli and a single-display setup is more suitable to observe the head orientation effect. The results in experiment 3 thus further confirmed that the zebrafish indeed spent more time looking at the upright BM stimuli rather than the inverted counterparts.

## 4. Discussion

The current study investigated an intriguing question whether fish, like land animals, are able to discriminate the upright BM of conspecifics from the inverted counterpart. Our results clearly showed that the zebrafish spent significantly more time dwelling on the area where the upright BM was displayed (experiments 1 and 2) and was more willing to watch the upright BM compared with the inverted BM (experiment 3). Taken together, the current study, to our knowledge, is the first demonstration of the inversion effect in BM perception in teleosts, a phenomenon that has already been found in humans, marmosets, baboons, and chicks [3–5, 7, 8, 10–14]. The findings thereby expand the applicability of the specialized “life motion detector” from terrestrial vertebrates to lower aquatic vertebrates and may further imply that this privileged ability of animacy detection is widely conserved in all vertebrates.

Consistent with the findings of Nakayasu and Watanabe [25], we found that the non-BM composed of 6 dots at equal distance with constant speed is much less attractive to fish. The fish could easily distinguish between the upright BM and the non-BM through the body configuration, swim kinetics, or swim trajectory [22, 26]. However, some previous studies found that the fish were similarly attracted by the upright and upside down real video clips of the conspecifics [31, 32], while the current study demonstrated that the zebrafish preferred to dwell on and orient towards the side of the screen displaying the upright relative to the inverted point-light BM. One possible reason for the discrepancy is that compared with the light-point BM stimuli, real video clips contain not only motion cues but also many other salient visual features including color, shape, and stripe pattern [21, 33, 34]. These distracting visual features do not change much with inversion and thus substantially increase the difficulty for the fish to distinguish the upright BM of the conspecifics from the inverted one. On the other hand, their selectively videoed motion clips (such as their exemplar demos) are probably short of crucial motion cues especially about the swimming trajectories in the vertical dimension, which may also lead to the decreased discriminability between the upright BM and the inverted BM. Considering that the only difference between the upright and the inverted point-light BM stimuli was the profile of the vertical motion velocity and acceleration (i.e., compatible or incompatible with the direction of gravity), it would be more difficult for fish to distinguish between the upright BM and the inverted BM than between the upright BM and the non-BM. Nevertheless, compared with the inverted BM, the fish were prone to spending more time near the upright BM in a multidisplay scene and more willing to orient towards the upright BM in a single-display scene. By and large, the zebrafish indeed can distinguish the upright point-light BM stimuli from the inverted counterparts, manifesting a robust inversion effect in BM perception.

The life detection ability of the zebrafish revealed by the current study echoes with those of the terrestrial animals in general. For terrestrial animals, the inversion effect in BM perception is contributed significantly by the dynamic motion cues [5, 10, 13, 35], as reviewed in Introduction that even scrambled motion cues without any global configural information are sufficient for the discrimination between the upright BM and the inverted BM. For teleosts, it has already been shown that biological postures, motion trajectories, and swimming kinetics all provide valuable clues to distinguish between constant motion and fish-like BM [22, 23, 25, 26]. In the current study, the fish BM stimuli consisted of a few dots with equidistance, and the inverted BM created by mirror flipping the upright BM vertically did not change the fish's postures (see the demos in Supplementary Materials). Accordingly, the observed inversion effect in BM perception in fish should be largely accounted for by motion dynamics rather than postures.

Regarding what kind of kinetic cues the “life motion detector” in terrestrial animals is attuned to, researchers propose that the gravity-dependent dynamics of life motion, especially arising from the feet motion, has unique advantages in visual processing [3, 4, 7, 11, 14]. Based on this, one possible explanation is that fish rely on the similar gravity-dependent dynamics of motion to perceive animacy, just like terrestrial vertebrates do. We therefore calculated the vertical accelerations and velocities of the BM stimuli from three fish and found a consistent pattern that the vertical accelerations of swimming up are slightly faster than swimming down, but their vertical velocities are a bit slower than swimming down. In other words, the subtle differences in motion dynamics between swimming up and swimming down may provide crucial clues for fish to perceive animacy (Table 1). It is conceivable that the movements of fish are also affected by the gravity of Earth because fish live in an environment where the difference in hydrostatic pressure is a direct product of gravity (Δ*P* = *ρg* Δ*h*, where *P*, *ρ*, *g*, and *h* represent the hydrostatic pressure, mass density, gravity, and height of vertical fluid column). More intriguingly, the similar pattern of the vertical accelerations also exists in the feet movements of human walkers and pigeons but not cats, which is consistent with the previous observation that the cat's feet motion carries the smallest inversion effect in comparison to other stimulus types [17]. These findings hint that at least the different magnitudes of vertical acceleration compatible or incompatible with the gravity of Earth may serve as valuable clues for most, if not all, vertebrates to perceive animacy conveyed by BM [20].

For terrestrial vertebrates, it has been hypothesized that the optic tectum (OT) and its mammalian equivalent, the superior colliculus (SC) [38, 39], are responsible for the early detection of BM via gravity-driven visual invariants derived from the upright feet motion [40, 41]. Given that the OT of teleosts has been revealed as one of the most prominent structures for visual motion information processing [42, 43], it may also be involved in distinguishing between the upright and inverted swimming kinetics of their conspecifics. The detection of gravitational acceleration in visual motion engages the vestibular network in humans [40], which is likely the case in zebrafish as well. Indeed, it has been shown that neurons in the zebrafish OT are involved in vestibular processing [44]. Therefore, the OT may play a crucial role of “life motion detector” in zebrafish.

On the other hand, as social animals, zebrafish prefer to shoal with their partners, but its underlying neural circuits remain largely unexplored. It has been suggested that the amygdala, septal, and preoptic areas are involved in many aspects of social behaviors in birds and mammals [45–47]. Particularly, the activations of the septal and preoptic areas have been observed when chicks are exposed to motion cues [45, 46]. The zebrafish brain regions including the dorsomedial and ventral telencephalon as well as the preoptic area are homologous to the mammalian amygdala, septum, and paraventricular nucleus of the hypothalamus, respectively [48]. Maybe these brain regions in zebrafish are also involved in BM perception. In many other species, such as humans and chicks, BM information is processed with apparent brain asymmetry [49, 50], but the evolutionary origin of which remains to be explored. What is more, it has been shown that lesion of the zebrafish ventral forebrain disrupts social orienting behaviors, indicating the indispensable role of the forebrain in social interaction [51]. In addition, oxytocin is a key neuropeptide for regulating social behaviors [52], and the expression of receptors affecting the release of oxytocin can modulate BM perception in zebrafish as well [23]. Interestingly, a specific effect of valproic acid (VPA) on dynamic cues of animate motion has been found in newly hatched chicks [53]. This means that VPA, which affects the social behaviors of zebrafish [54, 55], may also have a specific effect on BM perception. However, the mechanism of its influence is still unclear. Further investigations need to be carried out to determine and delineate the neural correlates of BM processing in zebrafish.

In sum, the current study demonstrates that zebrafish, similar to the terrestrial vertebrates, perceive BM in a gravity-dependent manner. The observed inversion effect in BM perception in zebrafish lends necessary support to the notion that the evolutionary origin and significance of the BM processing may be at least traced back to ancient vertebrates living in the ocean, or even to some invertebrates such as spiders [56]. Although the evidence so far provides an evolutionary possibility that the animacy detection ability is an ancient function inherited and developed for purposes of animal survival and social interaction, its underlying neural mechanism still awaits investigations by future research.

## Figures and Tables

**Table 1:** Average vertical accelerations and velocities for fish BM and the feet BM of humans and other animals. Positive and negative vertical acceleration and velocity values indicate upward and downward acceleration and velocity, respectively. Fish BM stimuli are from Nakayasu and Watanabe [25], while humans and other animal BM stimuli are from Vanrie and Verfaillie [36]^1^ and D. H. Chang and Troje [37]^2^, respectively.

Type	Vertical acceleration (a.u./s^2^)	Vertical velocity (a.u./s)
Fish	(+)734.53/(-)728.34	(+)34.76/(-)43.63
Fish	(+)533.10/(-)531.73	(+)23.11/(-)27.81
Fish	(+)573.37/(-)564.29	(+)23.33/(-)31.88
Human Walker^1^	(+)1721.30/(-)1406.30	(+)51.43/(-)47.25
Human Walker^2^	(+)3685.38/(-)3320.34	(+)335.26/(-)309.40
Pigeon^2^	(+)2738.10/(-)2691.70	(+)120.88/(-)230.13
Cat^2^	(+)2337.70/(-)2424.20	(+)139.27/(-)109.70

## Data Availability

All the data used for statistics, the code to generate the figures, and the demos can be found at http://ir.psych.ac.cn/handle/311026/42422.

## Supplementary Materials

Supplementary Materials Supplementary video 1 (Demo_UprightFishBM): an example of the upright BM stimulus created by capturing the motion of a freely swimming fish. Supplementary video 2 (Demo_InvertedFishBM): an example of the inverted BM stimulus created by mirror flipping the upright BM stimulus vertically. Supplementary video 3 (Demo_NonBMControl): an example of the non-BM stimulus created by six equally spaced dots moving along a linear trajectory with a constant speed.
