# Erratum to “Gravity-Dependent Animacy Perception in Zebrafish”

**DOI:** 10.34133/research.0265

**Published:** 2023-12-12

**Authors:** Xiaohan Ma, Xiangyong Yuan, Jiahuan Liu, Li Shen, Yiwen Yu, Wen Zhou, Zuxiang Liu, Yi Jiang

**Affiliations:** ^1^ State Key Laboratory of Brain and Cognitive Science, CAS Center for Excellence in Brain Science and Intelligence Technology, Institute of Psychology, Chinese Academy of Sciences, Beijing 100101, China.; ^2^ University of Chinese Academy of Sciences, Beijing 100049, China.; ^3^ Chinese Institute for Brain Research, Beijing 102206, China.; ^4^ State Key Laboratory of Brain and Cognitive Science, Institute of Biophysics, Chinese Academy of Sciences, Beijing 100101, China.; ^5^ Institute of Artificial Intelligence, Hefei Comprehensive National Science Center, Hefei 230088, China.

In the Research Article “Gravity-Dependent Animacy Perception in Zebrafish” [1], there was an error when calculating the velocity and acceleration of the human and pigeon BM stimuli provided by other researchers in Table. The error does not affect the main findings of the current study. The corrected table is shown below.

**Table.** Average vertical accelerations and velocities for fish BM and the feet BM of humans and other animals. Positive and negative vertical acceleration and velocity values indicate upward and downward acceleration and velocity, respectively. Fish BM stimuli are from Nakayasu and Watanabe [25], while humans and other animal BM stimuli are from Vanrie and Verfaillie [36]^1^ and H. Chang and Troje [37]^2^, respectively.

Type	Vertical acceleration (a.u./s^2^)	Vertical velocity (a.u./s)
Fish	(+)734.53/(−)728.34	(+)34.76/(−)43.63
Fish	(+)533.10/(−)531.73	(+)23.11/(−)27.81
Fish	(+)573.37/(−)564.29	(+)23.33/(−)31.88
Human Walker^1^	(+)1,675.71/(−)2,300.00	(+)78.75/(−)114.67
Human Walker^2^	(+)3,034.25/(−)3,365.53	(+)284.32/(−)321.64
Pigeon^2^	(+)5,046.55/(−)7,001.66	(+)384.62/(−)421.86
Cat^2^	(+)2,337.66/(−)2,424.22	(+)139.27/(−)109.70

Due to the error, several expressions (the underlined words below) in the published version should be revised accordingly.

(1) In the abstract, “More intriguingly, when the recorded point-light video clips of fish were directly compared with those of human walkers and pigeons, we could identify a unique and consistent pattern of accelerating movements in the vertical (gravity) direction.” It should read “More intriguingly, when the recorded point-light video clips of fish were directly compared with those of human walkers and pigeons, we could identify a unique and consistent pattern of movements in the vertical (gravity) direction.”

(2) In the discussion, “More intriguingly, the similar pattern of the vertical accelerations also exists in the feet movements of human walkers and pigeons but not cats, which is consistent with the previous observation that the cat’s feet motion carries the smallest inversion effect in comparison to other stimulus types [17]. These findings hint that at least the different magnitudes of vertical acceleration compatible or incompatible with the gravity of Earth may serve as valuable clues for most, if not all, vertebrates to perceive animacy conveyed by BM [20].” It should read “More intriguingly, the similar pattern of the vertical velocities also exists in the feet movements of human walkers and pigeons but not cats, which is consistent with the previous observation that the cat’s feet motion carries the smallest inversion effect in comparison to other stimulus types [17]. These findings hint that at least the different magnitudes of vertical velocity compatible or incompatible with the gravity of Earth may serve as valuable clues for most, if not all, vertebrates to perceive animacy conveyed by BM [20].”

These changes have been made in the corrected PDF and HTML (full text). Data and code for Table are available from the corresponding authors upon request.

## References

[1] Ma X, Yuan X, Liu J, Shen L, Yu Y, Zhou W, Liu Z, Jiang Y. Gravity-dependent animacy perception in zebrafish. Research. 2022;2022:9829016. 36128180 10.34133/2022/9829016 PMC9470206

